# (*E*)-2,2-Dimethyl-5-(3-phenyl­allyl­idene)-1,3-dioxane-4,6-dione

**DOI:** 10.1107/S1600536810042534

**Published:** 2010-10-23

**Authors:** Wu-Lan Zeng

**Affiliations:** a MicroScale Science Institute, Department of Chemistry and Chemical Engineering, Weifang University, Weifang 261061, People’s Republic of China

## Abstract

The title compound, C_15_H_14_O_4_, was prepared by the reaction of 2,2-dimethyl-1,3-dioxane-4,6-dione and (*Z*)-3-phenyl­acryl­aldehyde in ethanol. The dioxane ring is in a sofa conformation with the C atom bonded to the two methyl groups forming the flap. With the exception of the flap atom and the methyl group C atoms, all other non-H atoms are essentially planar, with an r.m.s. deviation of 0.067 (1) Å. The crystal structure is stabilized by weak inter­molecular C—H⋯O hydrogen bonds.

## Related literature

For background to Meldrum’s acid, 2,2-dimethyl-1,3-dioxane-4,6-dione, see: Kuhn *et al.* (2003 ▶); Casadesus *et al.* (2006 ▶). For a related structure, see: Zeng & Jian (2009 ▶).
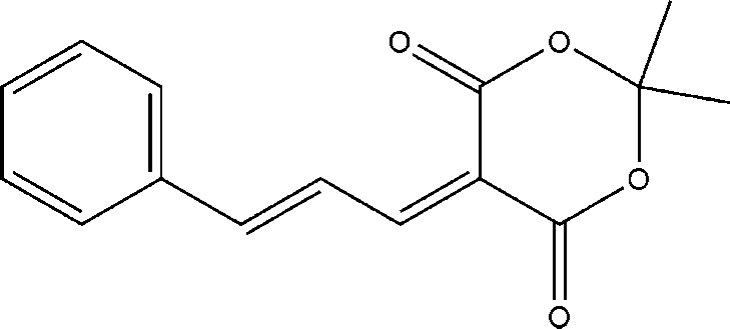


## Experimental

### 

#### Crystal data

C_15_H_14_O_4_
*M* *_r_* = 258.26
Triclinic, 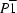

*a* = 6.9171 (14) Å
*b* = 7.0961 (14) Å
*c* = 13.732 (3) Å
α = 94.79 (3)°
β = 90.79 (3)°
γ = 98.31 (3)°
*V* = 664.4 (2) Å^3^
*Z* = 2
Mo *K*α radiation
μ = 0.09 mm^−1^
*T* = 293 K
0.20 × 0.15 × 0.10 mm

#### Data collection

Bruker SMART CCD area-detector diffractometer
6475 measured reflections
3006 independent reflections
2319 reflections with *I* > 2σ(*I*)
*R*_int_ = 0.045

#### Refinement

*R*[*F* ^2^ > 2σ(*F* ^2^)] = 0.055
*wR*(*F* ^2^) = 0.170
*S* = 1.25
3006 reflections
172 parameters
H-atom parameters constrained
Δρ_max_ = 0.33 e Å^−3^
Δρ_min_ = −0.30 e Å^−3^

### 

Data collection: *SMART* (Bruker, 1997 ▶); cell refinement: *SAINT* (Bruker, 1997 ▶); data reduction: *SAINT*; program(s) used to solve structure: *SHELXS97* (Sheldrick, 2008 ▶); program(s) used to refine structure: *SHELXL97* (Sheldrick, 2008 ▶); molecular graphics: *SHELXTL* (Sheldrick, 2008 ▶); software used to prepare material for publication: *SHELXTL*.

## Supplementary Material

Crystal structure: contains datablocks global, I. DOI: 10.1107/S1600536810042534/lh5147sup1.cif

Structure factors: contains datablocks I. DOI: 10.1107/S1600536810042534/lh5147Isup2.hkl

Additional supplementary materials:  crystallographic information; 3D view; checkCIF report

## Figures and Tables

**Table 1:** Hydrogen-bond geometry (Å, °)

*D*—H⋯*A*	*D*—H	H⋯*A*	*D*⋯*A*	*D*—H⋯*A*
C15—H15*B*⋯O3^i^	0.96	2.41	3.2991 (19)	155
C15—H15*C*⋯O3^ii^	0.96	2.57	3.486 (2)	159

Symmetry codes: (i) 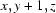; (ii) 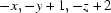.

**Fig. 1.**
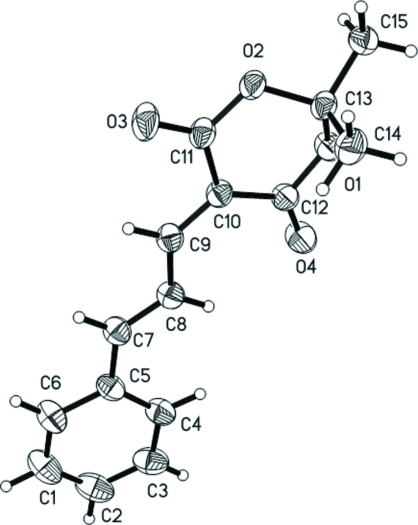
The molecular structure of (I), drawn with 30% probability ellipsoids and spheres of arbritrary size for the H atoms.

## supplementary crystallographic information

### Comment

Starting with its discovery and correct structural assignment, Meldrum's acid has become a widely used reagent in organic synthesis (Kuhn *et al.*, 2003; Casadesus *et al.*, 2006) owing to the interesting conformational features of the products. We have recently reported the crystal structure of 5-(2-fluorobenzylidene)-2,2-dimethyl-1,3-dioxane-4,6-dione (Zeng *et al.* 2009). As part of our search for new Meldrum's acid, the title compound (I) has been synthesized and its structure is reported herein. The molecular structure of (I) is shown in Fig. 1. The dioxane ring is in a  sofa conformation with the C atom bonded to the two methyl groups forming the flap. With the exception of the flap atom and the methyl group C atoms, all other non-hydrogen atoms are essentially planar with an rms deviation of 0.067 (1)Å. The deviation of atom C13 from the mean-plane formed by O1/O2/C11/C12/C10 is 0.270 (1)Å. The crystal structure is stabilized by weak intermolecular C—H···O hydrogen bonds (Table 1).

### Experimental

The mixture of malonic acid (6.24 g, 0.06 mol) and acetic anhydride(9 ml) in strong sulfuric acid (0.25 ml) was stirred with water at 303K, After dissolving, propan-2-one (3.48 g, 0.06 mol) was added dropwise into solution for 1 h. The reaction was allowed to proceed for 2 h. The mixture was cooled and filtered, and then an ethanol solution of (*Z*)-3-phenylacrylaldehyde (7.92g,0.06 mol) was added. The solution was then filtered and concentrated. Single crystals were obtained by evaporation of an petroleum ether-ethylacetate (4:1 *v*/*v*) solution of (I) at room temperature over a period of several days.

### Refinement

The H atoms were placed in calculated positions (C—H = 0.93 and 0.96 Å), and refined as riding with *U*_iso_(H) = 1.2*U*_eq_(C) or 1.5U_eq_(methyl C).

### Crystal data

**Table:**

C_15_H_14_O_4_	*Z* = 2
*M**_r_* = 258.26	*F*(000) = 272
Triclinic, *P*1	*D*_x_ = 1.291 Mg m^−^^3^
Hall symbol: -P 1	Mo *K*α radiation, λ = 0.71073 Å
*a* = 6.9171 (14) Å	Cell parameters from 2319 reflections
*b* = 7.0961 (14) Å	θ = 3.2–27.5°
*c* = 13.732 (3) Å	µ = 0.09 mm^−^^1^
α = 94.79 (3)°	*T* = 293 K
β = 90.79 (3)°	Block, yellow
γ = 98.31 (3)°	0.20 × 0.15 × 0.10 mm
*V* = 664.4 (2) Å^3^	

### Data collection

**Table:**

Bruker SMART CCD area-detector diffractometer	2319 reflections with *I* > 2σ(*I*)
Radiation source: fine-focus sealed tube	*R*_int_ = 0.045
graphite	θ_max_ = 27.5°, θ_min_ = 3.2°
phi and ω scans	*h* = −8→8
6475 measured reflections	*k* = −9→9
3006 independent reflections	*l* = −17→17

### Refinement

**Table:**

Refinement on *F*^2^	Primary atom site location: structure-invariant direct methods
Least-squares matrix: full	Secondary atom site location: difference Fourier map
*R*[*F*^2^ > 2σ(*F*^2^)] = 0.055	Hydrogen site location: inferred from neighbouring sites
*wR*(*F*^2^) = 0.170	H-atom parameters constrained
*S* = 1.25	*w* = 1/[σ^2^(*F*_o_^2^) + (0.1*P*)^2^] where *P* = (*F*_o_^2^ + 2*F*_c_^2^)/3
3006 reflections	(Δ/σ)_max_ < 0.001
172 parameters	Δρ_max_ = 0.33 e Å^−^^3^
0 restraints	Δρ_min_ = −0.30 e Å^−^^3^

### Special details

**Table:**

Geometry. All e.s.d.'s (except the e.s.d. in the dihedral angle between two l.s. planes) are estimated using the full covariance matrix. The cell e.s.d.'s are taken into account individually in the estimation of e.s.d.'s in distances, angles and torsion angles; correlations between e.s.d.'s in cell parameters are only used when they are defined by crystal symmetry. An approximate (isotropic) treatment of cell e.s.d.'s is used for estimating e.s.d.'s involving l.s. planes.
Refinement. Refinement of *F*^2^ against ALL reflections. The weighted *R*-factor *wR* and goodness of fit *S* are based on *F*^2^, conventional *R*-factors *R* are based on *F*, with *F* set to zero for negative *F*^2^. The threshold expression of *F*^2^ > σ(*F*^2^) is used only for calculating *R*-factors(gt) *etc*. and is not relevant to the choice of reflections for refinement. *R*-factors based on *F*^2^ are statistically about twice as large as those based on *F*, and *R*- factors based on ALL data will be even larger.

### Fractional atomic coordinates and isotropic or equivalent isotropic displacement parameters (Å^2^)

**Table:**

	*x*	*y*	*z*	*U*_iso_*/*U*_eq_	
O2	0.11039 (12)	0.49034 (12)	0.88464 (7)	0.0556 (3)	
O1	0.26632 (13)	0.73340 (13)	0.79292 (7)	0.0576 (3)	
C10	0.40643 (17)	0.44465 (17)	0.80181 (9)	0.0489 (3)	
C9	0.53406 (18)	0.32290 (19)	0.77683 (9)	0.0533 (3)	
H9A	0.5087	0.2047	0.8024	0.064*	
O4	0.52765 (15)	0.70212 (16)	0.70801 (9)	0.0790 (4)	
O3	0.21672 (17)	0.21587 (14)	0.89068 (9)	0.0782 (4)	
C11	0.24288 (19)	0.37290 (18)	0.86240 (9)	0.0534 (3)	
C8	0.70169 (18)	0.3474 (2)	0.71739 (10)	0.0546 (3)	
H8A	0.7361	0.4617	0.6891	0.066*	
C12	0.41250 (18)	0.63399 (19)	0.76415 (10)	0.0537 (3)	
C15	−0.0208 (2)	0.7761 (2)	0.87670 (11)	0.0621 (4)	
H15A	−0.0951	0.7213	0.8191	0.093*	
H15B	0.0082	0.9121	0.8745	0.093*	
H15C	−0.0953	0.7481	0.9335	0.093*	
C7	0.80960 (19)	0.2062 (2)	0.70229 (9)	0.0569 (3)	
H7A	0.7673	0.0944	0.7315	0.068*	
C5	0.98573 (19)	0.2077 (2)	0.64538 (9)	0.0553 (3)	
C13	0.16573 (17)	0.69346 (17)	0.88135 (9)	0.0503 (3)	
C4	1.06834 (19)	0.3651 (2)	0.59900 (11)	0.0648 (4)	
H4A	1.0124	0.4769	0.6049	0.078*	
C6	1.0760 (2)	0.0447 (2)	0.63695 (11)	0.0726 (4)	
H6A	1.0236	−0.0620	0.6680	0.087*	
C14	0.2935 (2)	0.7718 (2)	0.96872 (12)	0.0717 (4)	
H14A	0.4106	0.7137	0.9673	0.108*	
H14B	0.2248	0.7443	1.0273	0.108*	
H14C	0.3271	0.9077	0.9675	0.108*	
C3	1.2336 (2)	0.3567 (3)	0.54389 (13)	0.0784 (5)	
H3A	1.2869	0.4624	0.5123	0.094*	
C2	1.3187 (2)	0.1942 (3)	0.53569 (14)	0.0887 (6)	
H2A	1.4286	0.1886	0.4979	0.106*	
C1	1.2416 (3)	0.0393 (3)	0.58337 (14)	0.0903 (6)	
H1A	1.3018	−0.0700	0.5794	0.108*	

### Atomic displacement parameters (Å^2^)

**Table:**

	*U*^11^	*U*^22^	*U*^33^	*U*^12^	*U*^13^	*U*^23^
O2	0.0584 (5)	0.0430 (5)	0.0644 (6)	0.0006 (4)	0.0176 (4)	0.0074 (4)
O1	0.0619 (5)	0.0547 (5)	0.0613 (6)	0.0155 (4)	0.0176 (4)	0.0212 (4)
C10	0.0530 (6)	0.0473 (7)	0.0473 (6)	0.0056 (5)	0.0066 (5)	0.0115 (5)
C9	0.0593 (7)	0.0520 (7)	0.0500 (7)	0.0098 (5)	0.0023 (5)	0.0097 (5)
O4	0.0782 (7)	0.0716 (7)	0.0972 (8)	0.0208 (5)	0.0424 (6)	0.0429 (6)
O3	0.1022 (8)	0.0478 (6)	0.0891 (8)	0.0118 (5)	0.0430 (6)	0.0245 (5)
C11	0.0652 (7)	0.0439 (7)	0.0509 (7)	0.0035 (5)	0.0112 (6)	0.0082 (5)
C8	0.0546 (7)	0.0582 (7)	0.0525 (7)	0.0106 (5)	0.0027 (5)	0.0086 (5)
C12	0.0531 (6)	0.0531 (7)	0.0577 (7)	0.0087 (5)	0.0127 (5)	0.0180 (6)
C15	0.0607 (7)	0.0590 (8)	0.0679 (9)	0.0116 (6)	0.0127 (6)	0.0063 (6)
C7	0.0603 (7)	0.0609 (8)	0.0519 (7)	0.0148 (6)	0.0021 (6)	0.0098 (6)
C5	0.0556 (7)	0.0672 (8)	0.0463 (7)	0.0197 (6)	−0.0027 (5)	0.0050 (6)
C13	0.0567 (6)	0.0423 (6)	0.0510 (7)	0.0014 (5)	0.0104 (5)	0.0081 (5)
C4	0.0582 (7)	0.0763 (10)	0.0642 (8)	0.0191 (7)	0.0015 (6)	0.0144 (7)
C6	0.0820 (9)	0.0768 (10)	0.0665 (9)	0.0339 (8)	0.0090 (8)	0.0096 (8)
C14	0.0797 (9)	0.0637 (9)	0.0678 (9)	0.0026 (7)	−0.0088 (7)	−0.0016 (7)
C3	0.0592 (8)	0.1082 (14)	0.0715 (10)	0.0144 (8)	0.0096 (7)	0.0236 (9)
C2	0.0677 (9)	0.1327 (18)	0.0731 (10)	0.0386 (10)	0.0170 (8)	0.0082 (11)
C1	0.0914 (12)	0.1067 (14)	0.0844 (12)	0.0537 (11)	0.0177 (10)	0.0057 (10)

### Geometric parameters (Å, °)

**Table:**

O2—C11	1.3468 (15)	C7—C5	1.456 (2)
O2—C13	1.4415 (14)	C7—H7A	0.9300
O1—C12	1.3590 (15)	C5—C4	1.387 (2)
O1—C13	1.4362 (15)	C5—C6	1.389 (2)
C10—C9	1.3501 (17)	C13—C14	1.4992 (19)
C10—C11	1.4702 (18)	C4—C3	1.385 (2)
C10—C12	1.4746 (17)	C4—H4A	0.9300
C9—C8	1.4256 (19)	C6—C1	1.374 (2)
C9—H9A	0.9300	C6—H6A	0.9300
O4—C12	1.1959 (16)	C14—H14A	0.9600
O3—C11	1.2004 (15)	C14—H14B	0.9600
C8—C7	1.3375 (18)	C14—H14C	0.9600
C8—H8A	0.9300	C3—C2	1.366 (2)
C15—C13	1.4955 (17)	C3—H3A	0.9300
C15—H15A	0.9600	C2—C1	1.372 (3)
C15—H15B	0.9600	C2—H2A	0.9300
C15—H15C	0.9600	C1—H1A	0.9300
			
C11—O2—C13	119.15 (10)	C6—C5—C7	118.85 (14)
C12—O1—C13	119.85 (9)	O1—C13—O2	110.44 (10)
C9—C10—C11	116.66 (11)	O1—C13—C15	106.78 (10)
C9—C10—C12	123.71 (12)	O2—C13—C15	106.17 (10)
C11—C10—C12	119.36 (11)	O1—C13—C14	110.25 (11)
C10—C9—C8	130.02 (12)	O2—C13—C14	109.54 (10)
C10—C9—H9A	115.0	C15—C13—C14	113.55 (13)
C8—C9—H9A	115.0	C3—C4—C5	120.42 (15)
O3—C11—O2	118.16 (12)	C3—C4—H4A	119.8
O3—C11—C10	124.58 (13)	C5—C4—H4A	119.8
O2—C11—C10	117.22 (10)	C1—C6—C5	120.81 (17)
C7—C8—C9	120.10 (13)	C1—C6—H6A	119.6
C7—C8—H8A	119.9	C5—C6—H6A	119.6
C9—C8—H8A	119.9	C13—C14—H14A	109.5
O4—C12—O1	118.08 (11)	C13—C14—H14B	109.5
O4—C12—C10	126.04 (12)	H14A—C14—H14B	109.5
O1—C12—C10	115.79 (11)	C13—C14—H14C	109.5
C13—C15—H15A	109.5	H14A—C14—H14C	109.5
C13—C15—H15B	109.5	H14B—C14—H14C	109.5
H15A—C15—H15B	109.5	C2—C3—C4	120.37 (17)
C13—C15—H15C	109.5	C2—C3—H3A	119.8
H15A—C15—H15C	109.5	C4—C3—H3A	119.8
H15B—C15—H15C	109.5	C3—C2—C1	119.80 (16)
C8—C7—C5	127.42 (13)	C3—C2—H2A	120.1
C8—C7—H7A	116.3	C1—C2—H2A	120.1
C5—C7—H7A	116.3	C2—C1—C6	120.36 (16)
C4—C5—C6	118.21 (13)	C2—C1—H1A	119.8
C4—C5—C7	122.93 (13)	C6—C1—H1A	119.8

### Hydrogen-bond geometry (Å, °)

**Table:**

*D*—H···*A*	*D*—H	H···*A*	*D*···*A*	*D*—H···*A*
C15—H15B···O3^i^	0.96	2.41	3.2991 (19)	155
C15—H15C···O3^ii^	0.96	2.57	3.486 (2)	159

Symmetry codes: (i) *x*, *y*+1, *z*; (ii) −*x*, −*y*+1, −*z*+2.
